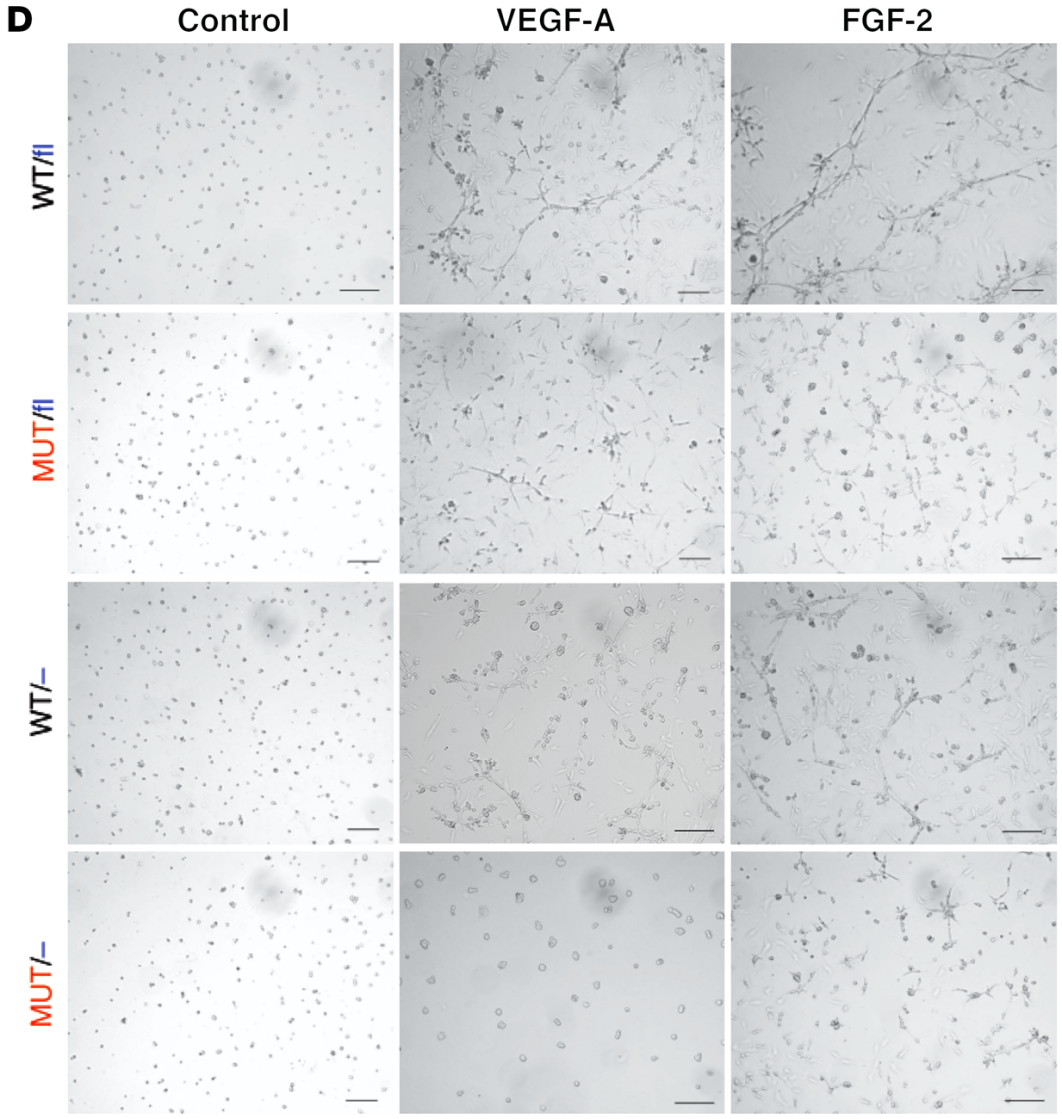# Corrigendum to RAS interaction with PI3K p110α is required for tumor-induced angiogenesis

**DOI:** 10.1172/JCI197925

**Published:** 2025-08-15

**Authors:** Miguel Manuel Murillo, Santiago Zelenay, Emma Nye, Esther Castellano, Francois Lassailly, Gordon Stamp, Julian Downward

Original citation: *J Clin Invest*. 2014;124(8):3601–3611. https://doi.org/10.1172/JCI74134

Citation for this corrigendum: *J Clin Invest*. 2025;135(16):e197925. https://doi.org/10.1172/JCI197925

In Figure 5D of the original article, the VEGF-A, WT/– image was incorrect. The authors determined that it was an image of the FGF-2, MUT/fl sample. The corrected figure panel, based on the original source data, is provided below. The HTML and PDF versions of the paper have been updated.

The authors regret the error.

## Figures and Tables

**Figure 5D:**